# Prescription opioid supply‐restricting policies and hospital use by people prescribed opioid medications, Victoria, 2018–22: a controlled interrupted time series analysis

**DOI:** 10.5694/mja2.52713

**Published:** 2025-06-26

**Authors:** Suzanne Nielsen, Louisa Picco, Bosco Rowland, Nadine E Andrew, Taya A Collyer, Samanta Lalic, Rachelle Buchbinder, Christopher Pearce, J Simon Bell, Dan I Lubman, Ting Xia

**Affiliations:** ^1^ Monash University Melbourne VIC; ^2^ Turning Point Alcohol and Drug Centre Eastern Health Melbourne VIC; ^3^ National Centre for Healthy Ageing Melbourne VIC; ^4^ Monash Health Melbourne VIC; ^5^ Outcome Health Melbourne VIC

**Keywords:** Policy, drugs and alcohol, Opioid‐related disorders, Pain management, Primary health care, Substance‐related disorders

## Abstract

**Objectives:**

To investigate the combined effect of two policies for reducing prescription opioid supply in Australia on hospital use by people prescribed opioids in primary care.

**Study design:**

Retrospective data linkage study; controlled interrupted time series analysis of linked primary care electronic medication records and hospital admissions data.

**Setting:**

Three Victorian health care networks (Monash Health, Eastern Health, Peninsula Health); pre‐intervention period: 1 April 2018 – 31 March 2020; intervention period: 1 April 2020 – 31 March 2022.

**Participants:**

People prescribed opioid medications at least twice during the preceding six months (opioid group) and propensity score‐matched patients, based on age, gender, comorbidity, and residential postcode‐based socio‐economic status (control group); matching was undertaken for each month of the study period.

**Intervention:**

Mandatory prescription drug monitoring (from 1 April 2020); tighter restriction criteria for the subsidisation of opioid medications by the Pharmaceutical Benefits Scheme (PBS) (from 1 June 2020).

**Main outcome measures:**

Differences between the opioid and control groups in immediate changes after start of the intervention in rates of emergency department (ED) presentation and hospital admission related to opioid use, non‐opioid substance use, self‐harm, or mental health problems; differences between the two groups in the change in trend for these rates between the pre‐intervention and intervention periods.

**Results:**

Propensity matching was undertaken for 179 091 people in the opioid group and a total of 389 061 people in the control group. The opioid‐related ED presentation rate for the opioid group had been increasing prior to the intervention, but declined after its introduction at a rate not significantly different from that of the control group. The immediate change in non‐opioid substance‐related ED presentation rate was greater for the opioid group than the control group (β, 11.1 [95% confidence interval, 1.7–20.5] presentations per 100 000 patients); by 31 March 2022, the rate had declined to below the pre‐intervention level. Differences between groups in changes to self‐harm‐ and mental health‐related presentations, and in all hospital admission rates, were not statistically significant.

**Conclusion:**

Following implementation of two prescription opioid supply‐restricting polices in 2020, the opioid‐related ED presentation rate declined among people prescribed opioids; the non‐opioid substance‐related presentation rate initially increased, but was lower than the pre‐intervention level by the end of the study period. Our findings suggest that some opioid‐restricting policies can reduce opioid‐related harm without increasing long term non‐opioid substance‐ or mental health‐related harm.

**Study registration:**

European post‐authorisation study register (EUROPAS), EUPAS104005 (prospective).

**The known**: Pharmaceutical opioids contribute to considerable harm in Australia. Many policies have been implemented in recent years to reduce high rates of opioid prescribing and related harm.

**The new**: Two opioid medication control policies implemented in mid‐2020 were associated with a decline in the opioid‐related emergency department presentation rate, but also with a sharp but temporary increase non‐opioid substance‐related presentations.

**The implications**: Policies that restrict opioid medication supply can reduce opioid‐related harm, but this outcome is accompanied by unintended consequences, including a short term increase in non‐opioid substance‐related harm.

The per capita opioid prescribing rate is higher in Australia than in the United States.^1^ In contrast to the United States, most opioid‐related deaths in Australia involve prescription opioids.^2^ Opioid‐related overdoses and deaths have prompted responses similar to those in other countries, including a national prescription drug monitoring program (in Victoria, an online check is required before prescribing monitored medicines)^3^ and changes to Pharmaceutical Benefits Scheme rules regarding subsidised opioids, including smaller pack sizes of opioids prescribed for treating acute pain, limits on long term opioid use, and more restricted indications for long term prescribing of opioids.^4^

In Australia, about 70% of opioids are prescribed in primary care, and most non‐fatal overdoses treated in emergency departments (EDs) and most opioid‐related deaths involve prescription opioids.^2^, ^5^ Restricting their supply can reduce the availability of opioids and reduce the number of deaths related to prescription opioids,^6^, ^7^, ^8^ but these restrictions can also hinder legitimate access to opioid medications, leading to unmanaged pain and distress.^9^

The possibility of a shift to riskier substances is also of concern.^10^, ^11^ In the United States, restricting the prescribing of opioids reduced prescribed opioid‐related harm but increased the number of illicit drug overdose deaths.^12^, ^13^ Opioid restrictions or discontinuation have also increased the incidence of opioid withdrawal symptoms (including psychological distress), uncontrolled pain, and suicide attempts.^14^, ^15^, ^16^ The effectiveness of policies for reducing prescription opioid supply and harms in Australia, and their unintended outcomes (eg, shifts to using other substances, harms related to reduced opioid access) have not been examined.

Hospitalisations and ED presentations have been key clinical outcomes in United States studies of the effect of opioid prescribing policies; ED presentations are a frequently used measure of acute drug poisonings.^17^, ^18^, ^19^ Few studies outside the United States have examined similar outcomes.

We therefore assessed the combined effect of two recent policies for reducing prescription opioid supply in Australia — mandatory prescription drug monitoring and tighter restriction criteria for the subsidisation of opioid medications by the Pharmaceutical Benefits Scheme (PBS) — on hospital use by people prescribed opioids in primary care. We investigated whether these changes had reduced opioid‐related harm, as indicated by opioid‐related ED presentation and hospital admission rates, and whether they were associated with unintended outcomes, such as higher rates of ED presentations and hospital admissions related to the use of non‐opioid substances and mental health problems (suicide, self‐harm, anxiety, depression).

## Methods

We undertook a retrospective analysis of linked primary care electronic medication records and hospital admissions data in Victoria for the period 1 April 2018 – 30 June 2022. The study protocol was published prospectively,^20^ and the study was prospectively registered with the European post‐authorisation study register (EUPAS104005; 7 August 2023). We report our analysis according to the Reporting of studies conducted using observational routinely collected health data statement for pharmacoepidemiology (RECORD‐PE), an extension of the STROBE and RECORD statements^21^ (Supporting Information).

### Data sources

Patient admission records with identifiers were provided by three Victorian health care networks (Monash Health, Eastern Health, Peninsula Health) with a combined catchment area that includes nearly 2.6 million people, 40% of the Victorian population.^22^ Using a set of three statistical linkage keys (SHA‐256 HASH keys), patient admission records were linked to the primary health care data collected by the Outcome Health Population Level Analysis and Reporting (POLAR) platform (Supporting Information, figure 1). Primary health care data were obtained from 562 general practices, or 55% of all practices in the health regions (the Eastern Melbourne, Gippsland, and Southeastern Melbourne primary health networks) that geographically correspond to the three health care networks.^23^, ^24^, ^25^ Opioid use in the three health regions corresponds to reported national opioid use;^22^, ^26^ 81% of admissions to hospitals in the three networks could be matched with primary care records. The linked databases include information about ED presentations, hospital admissions, patient demographic characteristics, recorded diagnoses, and medications prescribed in general practices (including both PBS‐subsidised medicines and those dispensed on private prescriptions). Data released to researchers were de‐identified and analysed on the Monash Secure eResearch Platform.

### Study periods

We assessed data for two study periods: the 24 months preceding the introduction of the mandatory prescription drug monitoring program (1 April 2018 – 31 March 2020); and the 22 months after both the mandatory monitoring and PBS opioid restrictions were introduced (1 June 2020 – 31 March 2022). The two‐month period during which the policies were introduced (1 April – 31 May 2020) was considered the intervention (Box 1). Linked data from before the study period were used to evaluate the historical medication use and comorbidity status of patients.

Box 1 **Study timeline**

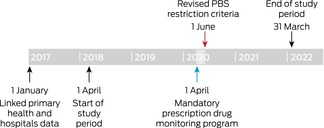

### Patient groups

We included data for people aged 14 years or older on 1 January 2017; as the data were available by five‐year age bands, we included data for people aged 14 years or older to ensured that data for people aged 18 years were captured, in accordance with our study protocol. We included people with Victorian residential postcodes for whom sufficient data for linkage keys were available (name and birth date), and who engaged in at least one activity at an included primary care practice during each of the two study periods, ensuring their active status before and after the introduction of the mandatory prescription drug monitoring program. We did not include people with cancer diagnoses at any point during the study, as the requirements of the prescription monitoring program and the changes in PBS subsidies did not target people with these diagnoses.

#### Opioid group

We included prescriptions of all opioid analgesics available in Australia during the study period (Supporting Information, table 1); prescriptions for opioid agonist treatment and cough medications (eg, high dose sublingual buprenorphine, methadone liquid, dihydrocodeine) were not included. For each month during the study period, the opioid group included people prescribed an opioid analgesic at least twice during the preceding six months, defined as recent opioid prescribing.

#### Control group

For people in the control group, no prescribing of opioids was recorded in the primary care dataset. Each patient in the opioid group was matched with one in the control group using propensity score matching. Propensity score matching improves the reliability of causal inferences by balancing covariates in intervention and control groups, reducing selection bias.^27^ The variables used for propensity matching were gender (based on socially constructed roles, behaviours, and identities: male or female), age, socio‐economic status measured using the Socioeconomic Indexes for Areas deciles (SEIFA) Index of Relative Socio‐economic Disadvantage (IRSD)^28^ derived from patient's postcode of residence, and comorbidity (Cambridge Multimorbidity Score, a weighted composite of 21 conditions;^29^ Supporting Information, table 2). The Cambridge Multimorbidity Score, a measure of long term health conditions in primary care patients, was applied as part of the matching process for people in the risk set for each month, based on information available at the start of the month. At the end of each month, people left both study groups and were re‐matched for the next month; the distribution of covariates was therefore balanced at each time point (standardised differences of less than 10%^30^). As individuals in the opioid group could be matched with different control group persons in different months, the total number of control group persons would be expected to exceed that of the opioid group.^31^

### Outcomes

The primary outcomes were the monthly rates (per 100 000 patients) of ED presentations and hospital admissions attributed to substance use or mental health problems, critical indicators influenced by changes in opioid prescribing.^14^, ^15^, ^32^ We defined the outcomes using International Statistical Classification of Diseases, tenth revision, Australian modification (ICD‐10‐AM) primary and secondary diagnosis codes (Supporting Information, table 3).

### Statistical analysis

All analyses were performed in Stata/MP 17. Changes in the monthly rate of each outcome after the introduction of the mandatory prescription drug monitoring program, were assessed in interrupted time series analyses, adjusted for baseline levels and temporal trends.^33^ We used a controlled interrupted time series analysis (ie, with a comparison group not exposed to the intervention) to reduce bias linked with underlying secular trends. Given the assumptions that underlie interrupted time series analysis, we assessed the data for stationarity and autocorrelation, and adjusted our analyses for coronavirus disease 2019 (COVID‐19) pandemic lockdown effects (Supporting Information, box 1).

In our models, step changes at selected timepoints indicate immediate changes in the outcome; the regression slope indicates the change in the outcome over time. We report the difference in the estimated regression coefficients (β; with 95% confidence interval, CI) between the opioid and control groups for the level change in each outcome with the introduction of the mandatory reporting program, and the difference (with 95% CI) between the two groups in the change in regression slope before and after the intervention. In a sensitivity analysis, we used a higher threshold for opioid group inclusion (at least four opioid analgesic prescriptions during the preceding six months).

### Ethics approval

The Monash Health Human Research Ethics Committee (project 76744: RES‐22‐0000‐026A; SSA/76744/MonH‐2021‐295413), Peninsula Health Human Research Ethics Committee (SSA/76744/PH‐2022), and Eastern Health Human Research Ethics Committee (S22‐032‐76744) approved the study.

## Results

Propensity matching was undertaken for 179 091 people in the opioid group and a total of 389 061 eligible people in the control group. Following propensity score matching, all covariates were well balanced at each time point (Box 2).

Box 2 Characteristics of the propensity‐matched opioid and control groups, April 2018*

**Table:** 

Characteristic	Opioid group	Control group	Standardised mean difference
Gender			0.0188
Female	34 671 (61.6%)	34 154 (60.7%)	
Male	21 604 (38.4%)	22 121 (39.3%)	
Age group (years)			0.0327
20–29	1575 (2.8%)	1615 (2.9%)	
30–39	4860 (8.6%)	4846 (8.6%)	
40–49	7476 (13.3%)	7233 (12.9%)	
50–59	10 303 (18.3%)	9913 (17.6%)	
60–69	10 709 (19.0%)	10 520 (18.7%)	
70–79	9443 (16.8%)	9653 (17.2%)	
80 or older	11 908 (21.2%)	12 494 (22.2%)	
Socio‐economic status, quintile^28^			0.0079
1 (most disadvantage)	7655 (13.6%)	7693 (13.7%)	
2	5202 (9.2%)	5244 (9.3%)	
3	13 516 (24.0%)	13 335 (23.7%)	
4	13 844 (24.6%)	13 853 (24.6%)	
5 (least disadvantage)	16 058 (28.5%)	16 150 (28.7%)	
Cambridge Multimorbidity Score, mean (SD)	1.83 (0.85)	1.85 (0.85)	0.0195

SD = standard deviation.

* Propensity score matching was conducted using the nearest neighbour algorithm, matching each person in the opioid group with one in the control group. This is a sample table, for April 2018; for our analysis, propensity matching was separately undertaken for each month of the study period.

### Emergency department presentations

In April 2018, the opioid‐related ED presentation rates for the two groups were not significantly different (opioid *v* control group: β, 3.68 [95% CI, –0.68 to 8.04] presentations per 100 000 patients). Prior to the intervention, the rate of change was greater for the opioid group than the control group (β, 0.37 [95% CI, 0.03 to 0.70] presentations per 100 000 patients per month); the slope difference after the intervention was not statistically significant (β, –0.23 [95% CI, –0.80 to 0.33] presentations per 100 000 per month). The presentation rate for the opioid group was increasing before the intervention, but declined after its introduction (Box 3; Box 4, panel A).

In April 2018, the non‐opioid substance‐related ED presentation rate was higher for the opioid than the control group (β, 9.40 [95% CI, 0.66 to 18.1] presentations per 100 000 patients), and the level change in presentation rate with the intervention was also greater for the opioid group (β, 11.1 [95% CI, 1.7 to 20.5] presentations per 100 000 patients). The slope difference between the two groups was statistically significant neither before nor after the intervention. By 31 March 2022, the rate for the opioid group had declined to below the estimated pre‐intervention level (Box 3; Box 4, panel B).

For self‐harm‐related ED presentations, neither the level changes in rates nor the slope change in presentation rate during the intervention period were significantly different between the two study groups (Box 3; Box 4, panel C). For mental health‐related ED presentations, neither the level changes in rates nor the slope change in presentation rate during the intervention period were significantly different between the two study groups (Box 3; Box 4, panel D).

### Hospital admissions

In April 2018, the opioid‐related hospital admission rate was higher for the opioid than the control group (β, 20.8 [95% CI, 12.5 to 29.2] admissions per 100 000 patients). Neither the level changes in rates (opioid *v* control group: β, 5.42 [95% CI, –5.28 to 16.1] admissions per 100 000 patients) nor the slope change during the intervention period (opioid *v* control group: β, –0.15 [95% CI, –0.95 to 0.64] admissions per 100 000 patients per month) were significantly different between the two study groups (Box 3; Box 5, panel A).

In April 2018, the non‐opioid substance‐related ED presentation rate was higher for the opioid than the control group (opioid *v* control group: 45.9 [95% CI, 33.0 to 58.9] admissions per 100 000 patients), and the rate of change in admission rate was greater (opioid *v* control group: –1.26 [95% CI, –2.25 to –0.27] admissions per 100 000 rate during the intervention period were significantly different between the two study groups (Box 3; Box 5, panel B).

In April 2018, the self‐harm‐related hospital admission rate was higher for the opioid than the control group (β, 19.7 [95% CI, 9.92 to 29.5] admissions per 100 000 patients); the rates of change in admission rate prior to program introduction were not significantly different. Neither the level changes in rates nor slope change during the intervention period were significantly different between the two study groups; the slope declined for the opioid group, but not for the control group, after the intervention (Box 3; Box 5, panel C).

In April 2018, the mental health‐related hospital admission rate was higher for the opioid than the control group (β, 78.0 [95% CI, 57.1 to 98.8] admissions per 100 000 patients); the rate of change in admission rate (decline) prior to the intervention was also greater for the opioid group (β, –1.92 [95% CI, –3.51 to –0.33] admissions per 100 000 patients per month). Neither the level changes nor the slope changes during the intervention period were significantly different between the two study groups. The modelled admission rate for the opioid group was below the level predicted by the pre‐intervention trend at the end of the study period; this difference was not statistically significant in the control group (Box 3; Box 5, panel D).

Box 3 The effect of the 2020 prescription opioid regulatory changes on emergency department presentations and hospital admissions of people in the catchment areas of three Victorian health care networks: comparison of patients prescribed opioid medications during the previous six months and propensity score‐matched primary care patients*

**Table:** 

	Coefficient (β) (95% confidence interval)			
Comparison (opioid *v* control group)	Opioid‐related	Non‐opioid substance‐related	Self‐harm‐related	Mental health‐related
**Emergency department presentations**				
Pre‐intervention				
Initial monthly rate (per 100 000 patients)	3.68 (–0.68 to 8.04)	**9.40 (0.66 to 18.1)**	1.56 (–5.16 to 8.33)	12.3 (–7.50 to 32.2)
Rate change (per 100 000 patients per month)	0.37* (0.03 to 0.70)	–0.34 (–0.92 to 0.23)	0.10 (–0.47 to 0.66)	–0.56 (–1.97 to 0.85)
Post‐intervention				
Immediate changes in monthly rate (per 100 000 patients)	–6.18 (–14.5 to 2.10)	11.1 (1.71 to 20.5)	–1.68 (–15.1 to 11.7)	–2.58 (–25.3 to 20.1)
Slope change (per 100 000 patients per month)	–0.23 (–0.80 to 0.33)	–0.22 (–1.0 to 0.56)	0.06 (–0.80 to 0.92)	0.84 (–0.92 to 2.60)
**Hospital admissions**				
Pre‐intervention				
Initial monthly rate (per 100 000 patients)	20.8* (12.5 to 29.2)	**45.9 (33.0 to 58.9)**	**19.7 (9.9 to 29.5)**	**78.0 (57.1 to 98.8)**
Rate change (per 100 000 patients per month)	–0.28 (–0.91 to 0.34)	**–1.26 (–2.25 to –0.27)**	–0.63 (–1.34 to 0.08)	**–1.92 (–3.51 to –0.33)**
Post‐intervention				
Immediate changes in monthly rate (per 100 000 patients)	5.42 (–5.28 to 16.1)	16.9 (–3.71 to 37.4)	2.09 (–12.7 to 16.9)	11.4 (–30.4 to 53.2)
Slope change (per 100 000 patients per month)	–0.15 (–0.95 to 0.64)	0.50 (–1.13 to 2.13)	1.07 (–0.00 to 2.15)	2.36 (–0.51 to 5.22)

Bold: Statistically significant.

* The pre‐intervention rate change (control group only), the immediate post‐intervention change, post‐intervention slope change (for each group), the level change at the end of the study period (for each group), and the between‐group difference in level at the end of the study period are provided in the Supporting Information, table 4 (emergency department presentations) and Supporting Information, table 5 (hospital admissions).

Box 4 Emergency department presentations, April 2018 – April 2022, by study group and month*

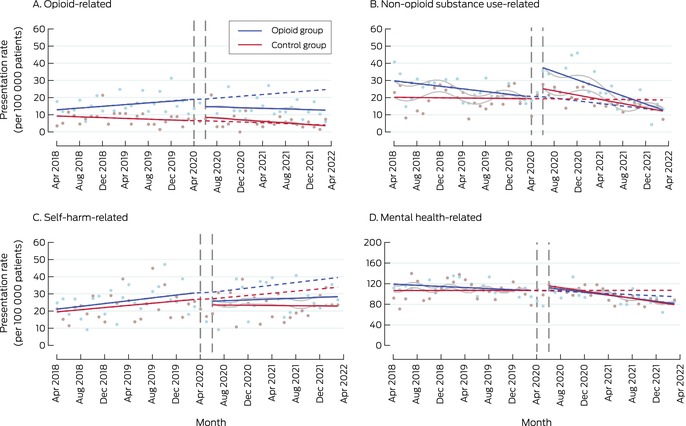

* Solid lines: regression curves by study period (before and after the intervention); dashed lines: predicted outcomes for the intervention period, based on the pre‐intervention trend; grey lines: regression curves for intervention period adjusted for coronavirus 2019 disease‐related lockdowns and seasonality.

Box 5 Hospital admissions, April 2018 – April 2022, by study group and month*

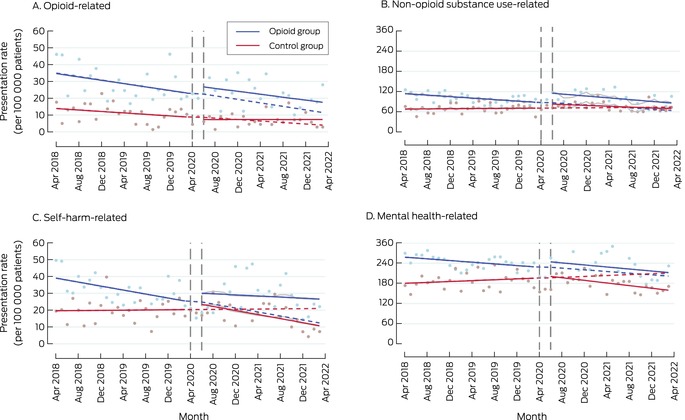

* Solid lines: regression curves by study period (before and after the intervention); dashed lines: predicted outcomes for the intervention period, based on the pre‐intervention trend; grey lines: regression curves for intervention period adjusted for coronavirus 2019 disease‐related lockdowns and seasonality.

### Sensitivity analysis

In the sensitivity analysis, 92 169 people were included in the opioid group (at least four opioid analgesic prescriptions during the preceding six months) and 258 301 eligible patients in the control group. Differences between the two study groups in neither immediate changes in rates nor the slope change during the intervention period were statistically significant for ED presentations or hospital admissions for any of the four causes (Supporting Information, table 6).

## Discussion

The introduction of the mandatory prescription drug monitoring program and revised PBS restriction criteria for opioid medications was followed by a decline in the opioid‐related ED presentation rate and an initial increase in that of non‐opioid substance‐related ED presentations among people prescribed opioid medications in three Victorian health care network catchment areas; the mental health‐related ED presentation rate did not change.

We report the first study outside the United States to examine hospital use by people prescribed opioid medications before and after prescription opioid policy changes, and our findings are broadly consistent with those reported there.^11^, ^34^ As we expected, the new policies were associated with reduced opioid‐related harm; the reduced availability of prescription opioids presumably explains this change.^8^, ^35^, ^36^ We also expected that the changes would have unintended effects, and found an immediate increase in the non‐opioid substance‐related ED presentation rate that was greater among people prescribed opioids than in the control group. One possible mechanism underlying the increase in non‐opioid substance‐related harms could be related to increased prescribing of unmonitored pain medicines, such as pregabalin and tricyclic antidepressants, following the introduction of the prescription drug monitoring program.^35^ Other possible mechanisms underlying increased non‐opioid substance‐related harm could be substitution effects; that is, using non‐opioid medications instead of opioids. We found evidence of these unintended effects only in the short term, with only limited evidence of longer term negative effects. As this is the first study of this type in a health care system outside the United States, our findings are of international relevance, and indicate that measuring a comprehensive range of outcomes is important when evaluating opioid policies.^36^

As people with acute drug‐related problems are often managed in EDs, without being admitted to hospital, ED presentations are a more sensitive measure of changes in harm levels, and our findings are consistent with this view. Our results also indicate the importance of continuing to study how prescription opioid restriction can be achieved in a manner that reduces opioid‐related harm while minimising other, unintended consequences. Specifically, it is important to understand which features could increase the effectiveness of prescription opioid restrictions. For example, the prescription drug monitoring program is mandatory in Victoria, access to the system is limited to health care providers (ie, excluding law enforcement authorities), and education was provided to health care providers, all of which are important for the outcomes of prescription drug monitoring programs.^37^, ^38^

### Limitations

We analysed linked primary care and hospitals data to examine the effect of opioid restriction policies on people prescribed opioids in primary care, with a control group matched according to key demographic and health variables. The inclusion of three large health regions provided geographic and socio‐economic diversity, with nationally representative opioid use patterns.^22^, ^26^ The inclusion of private (eg, immediate release tapentadol) and subsidised opioid prescriptions facilitated more comprehensive analysis of opioid prescribing. The use of individual patient‐level privacy‐preserving identifiers through statistical linkage keys also enabled identification of the same person across multiple practices. However, we could not distinguish between illicit and prescribed opioids in ED and hospital data because of the frequent use of non‐specific opioid poisoning codes in these datasets; as in similar studies, we therefore examined overall opioid‐related harm.^34^, ^39^ We must also be cautious because the period between the introduction of mandatory monitoring and the revised PBS criteria coincided with a period of COVID‐19‐related restrictions. However, we controlled for lockdown periods in our analyses, and we included a control group, thereby limiting the potential for bias and increasing our ability to draw conclusions about policy effects. Although the illicit drug market was affected by COVID‐19‐related restrictions, the price and population‐level consumption of heroin in Victoria were relatively stable.^40^ The reduced mental health‐related admission rates for both study groups were consistent with other reports regarding the COVID‐19 restrictions period.^41^ Nevertheless, further studies of similar policy changes implemented outside the COVID‐19‐related restrictions period are warranted.

### Conclusion

We found that, among people prescribed opioid medications in primary care, the opioid‐related ED presentation rate was reduced and that of non‐opioid substance‐related ED presentations increased in the short term after the introduction of mandatory prescription drug monitoring and revised PBS restriction criteria for opioid medications in 2020. These initiatives did not affect rates of hospitalisations related to opioid or non‐opioid substance use or mental health problems. Our findings indicate that a health‐focused approach to prescription drug monitoring could avoid some of the harms reported in the United States. As similar opioid restriction policies are implemented across Australia and elsewhere, being aware of these outcomes will be important.

## Open access

Open access publishing facilitated by Monash University, as part of the Wiley – Monash University agreement via the Council of Australian University Librarians.

## Competing interests

No relevant disclosures.

## Data sharing

The datasets provided for this study cannot be shared because of the conditions in the data sharing agreements with data custodians. Researchers wishing to access Eastern Melbourne, Gippsland, or South Eastern Melbourne Primary Heath Network, data should contact Outcome Health (POLAR data custodians; admin@outcomehealth.org.au). Those wishing to access Monash Health, Eastern Health and Peninsula Health should contact the respective health services.

## Authors’ contributions

All authors contributed to the study's conception and design. Ting Xia cleaned the data, derived the interrupted time series models, and performed the analysis. Bosco Rowland, Nadine Andrew, and Taya Collyer provided critical insight into the analytical measures and approaches. Suzanne Nielsen and Ting Xia took the lead in writing the manuscript. All authors provided critical feedback and helped shape the research, analysis and manuscript.

Received 6 August 2024, accepted 21 January 2025.

## Supporting information


Supplementary methods and results
