# Supplementary material for: Prescription opioid supply‐restricting policies and hospital use by people prescribed opioid medications, Victoria, 2018–22: a controlled interrupted time series analysis
Source: Med J Aust. 2025 Jun 26;223(3):134–40. doi: 10.5694/mja2.52713 (PMC12318491; doi:10.5694/mja2.52713)
Supplement: Supplementary file 1 — Supplementary methods and results [file MJA2-223-134-s001.pdf]

The Medical Journal of Australia

Supporting Information

Supplementary methods and results

This appendix was part of the submitted manuscript and has been peer reviewed.
It is posted as supplied by the authors.

Appendix to: Nielsen S, Picco L, Rowland B, et al. Prescription opioid supply-restricting policies and hospital use by people prescribed opioid medications, Victoria, 2018–22: a controlled interrupted time series analysis. *Med J Aust* 2025; doi: 10.5694/mja2.52713.

Supplementary methods

Figure 1. Data linkage flow

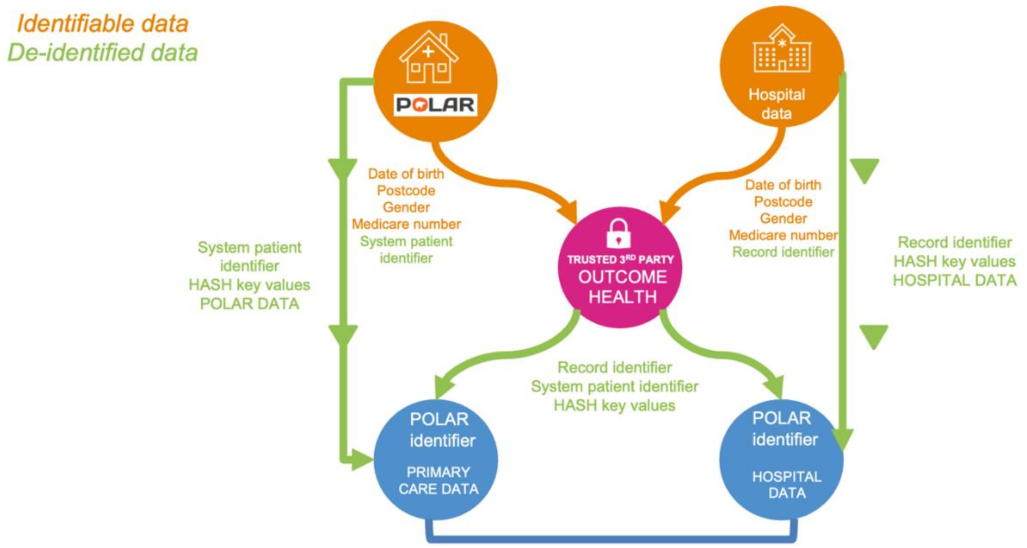

POLAR (POpulation Level Analysis and Reporting), is a web-based business intelligence tool automatically extracts patient data from the general practice clinical information software and practice management software. Outcome Health (https://www.outcomehealth.org.au) is a not-for-profit organisation that provides a range of dedicated data intelligence and clinical services needed to help Australia's health system further improve patient outcomes in all communities.

Table 1. Included opioids and Anatomical Therapeutic Chemical (ATC) codes

**Opioid type**	**Anatomical Therapeutic Chemical (ATC) classification code**
Codeine	R05DA04
Codeine combinations	
Aspirin/codeine	N02AJ07
Ibuprofen/codeine	N02AJ08
Paracetamol/codeine	N02AA59, N02AJ06
Paracetamol/codeine/doxylamine	N02AA59, N02AJ06
Fentanyl	N02AB03, N01AH01
Buprenorphine	N02AE01
Dextropropoxyphene	N02AC54
Methadone	N02AC52, N07BC02
Morphine	N02AA01
Oxycodone	N02AA05
Oxycodone/naloxone	N02AA55
Hydromorphone	N02AA03
Tapentadol	N02AX06
Tramadol and paracetamol/tramadol	N02AX02, N02AJ13
Pethidine	N02AB02

Table 2. Modified Cambridge Multimorbidity Score (CMMS) using electronic health record (Systematized Nomenclature of Medicine-Clinical Terms (SNOMED CT)

Condition	Observation type	Calculation type
Alcohol Problems	≥ 1 x Diagnosis	Ever
Anxiety or Depression	≥ 1 x Diagnosis (ever recorded)<br>or<br>≥ 2 x Prescriptions in the last 12 months	
Atrial Fibrillation	≥ 1 x Diagnosis	Ever
Cancer	≥ 1 x Diagnosis	Ever
Chronic Kidney Disease	Pathology results	See footnote*
Chronic Liver Disease and Viral Hepatitis	≥ 1 x Diagnosis	Ever
Constipation	≥ 4 x Prescriptions	Last 12 months
Chronic Obstructive Pulmonary Disease	≥ 1 x Diagnosis	Ever
Dementia	≥ 1 x Diagnosis	Ever
Diabetes	≥ 1 x Diagnosis	Ever
Disorder of Prostate	≥ 1 x Diagnosis	Ever
Epilepsy	≥ 1 x Diagnosis (ever recorded)<br>and<br>≥ 1 x Prescriptions in the last 12 months	
Heart Failure	≥ 1 x Diagnosis	Ever
Irritable Bowel Syndrome	≥ 1 x Diagnosis (ever recorded)<br>or<br>≥ 4 x Prescriptions in the last 12 months	
Learning Disability	≥ 1 x Diagnosis	Ever
Multiple Sclerosis	≥ 1 x Diagnosis	Ever
Painful Condition#	≥ 4 x Prescriptions in the last 12 months	
Parkinsonism	≥ 1 x Diagnosis	Ever
Peripheral Vascular Disease in the Leg	≥ 1 x Diagnosis	Ever
Psychoactive Substance Misuse	≥ 1 x Diagnosis	Ever
Schizophrenia or Bipolar Disorder	≥ 1 x Diagnosis (ever recorded)<br>or<br>≥ 1 x Prescriptions in the last 12 months	

Ever applies to whether this diagnosis was ever recorded.
Published CMMS weights were applied to the 21 comorbidities to calculate the final multimorbidity score for each person. * People were considered to have chronic kidney disease if the average of the two most recent estimated glomerular filtration rates (eGFRs) recorded prior to the index date was < 60mL/min.
Some prescriptions (e.g. gabapentin, pregabalin) were counted as an eligible prescription if there was no diagnosis of epilepsy, consistent with the original CMMS rules by Tsang et al [1].

Table 3. ICD-10-AM diagnosis codes used to define mental health-related hospital separations

Primary and secondary diagnoses related to ED presentations and hospital attendance were coded according to the International Statistical Classification of Diseases and Related Health Problems, Tenth Revision, Australian Modification (ICD-10-AM). Opioid-related presentations were identified where the primary or additional diagnoses include (1) F11.0-F11.9 (opioid-related disorders) and (2) T40.0-T40.4, T40.6 (poisoning by, adverse effects of, and underdosing of narcotics and psychodysleptics).

Non–opioid-related substance use included substances such as alcohol, heroin, and cannabis identified by the ICD-10-AM codes F10, F12-F19. Cases of suicide attempts and self-harm that satisfy the following criteria included: (1) a principal diagnosis in the ICD-10-AM range S00-T75, T79 (injury, poisoning, and certain other consequences of external causes); (2) the first reported external cause code in the record in the ICD-10-AM range X60-X84, Y87.0 (external causes of morbidity); and (3) suicide-related behaviors (R45.81 [suicidal ideation]). Presentations and admissions for other mental health conditions were identified using a modified set of codes from the National Hospital Morbidity Database's criteria for mental health-related hospital separations.

ICD-10 Codes	Description
F00-F09	Organic, including symptomatic, mental disorders
F20-F29	Schizophrenia, schizotypal and delusional disorders
F30-F39	Mood (affective) disorders
F40-F48	Neurotic, stress-related and somatoform disorders
F50-F59	Behavioural syndromes associated with physiological disturbances and physical factors
F60-F69	Disorders of adult personality and behaviour
F80-F89	Disorders of psychological development
F99	Unspecified mental disorder
G47	Sleep disorders
O99.3	Mental disorder nervous system pregnancy and birth
R44	Other symptoms and signs involving general sensations and perceptions
R45.0	Nervousness
R45.1	Restlessness and agitation
R45.4	Irritability and anger
Z00.4	General psychiatric examination, not elsewhere classified
Z03.2	Observation for suspected mental and behavioural disorder
Z04.6	General psychiatric examination, requested by authority
Z13.3	Special screening examination for mental and behavioural disorders
Z50.2	Alcohol rehabilitation
Z50.3	Drug rehabilitation
Z63.1	Problems relationship w parents & in-laws
Z63.8	Other spec problems related to prim support group
Z63.9	Problem related to primary support group, unspecified
Z65.8	Other specified problems related to psychosocial circumstances
Z65.9	Problem related to unspecified psychosocial circumstances
Z71.4	Counselling and surveillance for alcohol use disorder
Z71.5	Counselling and surveillance for drug use disorder

Box 1. Interrupted time series analysis: supplementary details

Model coding

Controlling for secular trends was done using the *itsa* software package which is an add-on for Stata statistical software package [1]. This package allows comparison of intercept and slope between groups at the first time point and subsequent time points. Thus, allowing to examine whether there are differences between the groups at the start of the study period and at the various policy intervention points.

Stationarity, autocorrelation, and seasonality testing

Newey–West standard errors for coefficients were produced to estimate by ordinary least squared regression. Stationarity of time series were assessed prior to modelling. Visual inspection indicated a potential trend, confirmed by the Augmented Dickey-Fuller test suggesting stationarity.

Autocorrelation was assessed using the Cumby-Huizinga test, testing the null hypothesis that the disturbance follows a moving average process up to order q (using the actest or actest robust commands in Stata 17 to account for conditional heteroskedasticity) [2]. We evaluated autocorrelation up to 12 lags. Based on results, lags of the dependent variable were incorporated into models, to address autocorrelation.

Following Jebb et al. [3] to address potential seasonality, we incorporated sine and/or cosine terms for each calendar month. These terms are smooth and symmetric over time, making them suitable for detecting consistent seasonal patterns of increase and decrease. After fitting initial models including these terms, we retained only those with $p < 0.05$ in final models.

Covariate

As Victorian coronavirus disease 2019 (COVID-19) related lockdown periods from May 2020 to October 2021 could impact hospital attendances[4], our model was also adjusted to account for the number of days under lockdown at each monthly data point. Regression models were fitted using 46 monthly rates of ED presentations and hospital admissions per 100,000 people for both the opioid and control groups (24 months before mandatory prescription drug monitoring program implementation, allowing a 2-month policy window and 22-months post implementation of changed opioid subsidies).

Supplementary results

Table 4. Interrupted time-series analysis: emergency department presentations

	Coefficient (β) (95% confidence interval)			
**Characteristic**	**Opioid-related**	**Non-opioid substance-related**	**Self-harm-related**	**Mental health-related**
**Pre-intervention**				
Pre-intervention trend (control group)	–0.12 (–0.30 to 0.07)	–0.16 (–0.54 to 0.23)	0.31 (–0.03 to 0.66)	–0.07 (–1.35 to 1.21)
Difference in the initial step of monthly rates per 100,000 (opioid v control group)	3.68 (–0.68 to 8.04)	9.40 (0.66 to 18.13)	1.59 (–5.16 to 8.33)	12.3 (–7.50 to 32.2)
Difference in the trends (slope) (opioid v control group)	**0.37 (0.03 to 0.70)**	–0.34 (–0.92 to 0.23)	0.10 (–0.47 to 0.66)	–0.56 (–1.97 to 0.85)
**Post-intervention**				
Step change (control group)	1.56 (–3.61 to 6.73)	**9.21 (3.36 to 15.05)**	–2.43 (–11.41 to 6.54)	13.9 (–5.20 to 33.2)
Step change (opioid group)	–4.62 (–11.0 to 1.81)	**20.3 (12.3 to 28.4)**	–4.11 (–14.8 to 6.60)	11.4 (–3.30 to 26.1)
Difference in the step changes in monthly rates per 100,000 immediately following the policy changes (opioid v control group)	–6.18 (–14.5 to 2.10)	**11.1 (1.71 to 20.5)**	–1.68 (–15.1 to 11.7)	–2.58 (–25.3 to 20.1)
Trends change (slope) (control group)	–0.10 (–0.45 to 0.25)	**–0.61 (–1.09 to –0.12)**	–0.39 (–0.93 to 0.16)	**–1.86 (–3.29 to –0.43)**
Trends change (slope) (opioid group)	–0.33 (–0.76 to 0.10)	**–0.83 (–1.44 to –0.22)**	–0.33 (–1.00 to 0.35)	–1.02 (–2.09 to 0.05)
Difference in changes in trends (slope) before and after the policy changes (opioid v control group)	–0.23 (–0.80 to 0.33)	–0.22 (–1.01 to 0.56)	0.06 (–0.80 to 0.92)	0.84 (–0.92 to 2.60)
Level change at the end study period [a] (opioid group)	**–12.4 (–21.8 to –2.93)**	2.41 (–14.06 to 18.9)	–10.94 (–28.0 to 6.13)	3.56 (–36.1 to 43.2)
Level change at the end study period (control group)	–0.53 (–9.91 to 1.90)	–4.9 (–18.7 to8.96)	–10.72 (–23.56 to 2.12)	–23.23 (–71.26 to 24.7)
Difference in difference overall effect [b] (opioid v control group)	**–11.8 (–11.9 to –4.83)**	**7.30 (4.68 to 9.92)**	–0.22 (–4.45 to 4.01)	**26.8 (35.0 to 18.5)**
Post-intervention linear trend (control group)	–0.21 (–0.52 to 0.09)	–0.07 (–0.50 to 0.36)	**–0.76 (–1.06 to –0.47)**	**–1.93 (–2.53 to –1.33)**
Post-intervention linear trend (opioid group)	–0.08 (–0.42 to 0.25)	0.08 (–0.43 to 0.59)	**–1.33 (–1.78 to –0.88)**	**–1.65 (–2.52 to –0.78)**

[a] Level change at end of study period and predicted level from control period trends.

[b.] Difference between level at end of study period and predicted level from control period trends.

Blue: results included in Box 3 of main article.

Table 5. Interrupted time-series analysis: hospital admissions

	Coefficient (β) (95% confidence interval)			
**Characteristic**	**Opioid-related**	**Non-opioid substance-related**	**Self-harm-related**	**Mental health-related**
**Pre-intervention**				
Pre-intervention trend: control	–0.22 (–0.50 to 0.06)	–0.02 (–0.75 to 0.71)	0.03 (–0.38 to 0.45)	0.66 (–0.64 to 1.96)
Difference in the initial step of monthly rates per 100,000 Opioid v control group	**20.8 (12.5 to 29.2)**	**45.9 (33.0 to 58.9)**	**19.7 (9.92 to 29.5)**	**78.0 (57.1 to 98.8)**
Difference in the trends (slope) Opioid v control group	–0.28 (–0.91 to 0.34)	**–1.26 (–2.25 to –0.27)**	–0.63 (–1.34 to 0.08)	**–1.92 (–3.51 to –0.33)**
**Post-intervention**				
Step change (control group)	–1.28 (–6.43 to 3.86)	**21.8 (6.15 to 37.5)**	2.02 (–6.46 to 10.5)	7.02 (–26.9 to 40.9)
Step change (opioid group)	4.33 (–4.65 to 13.3)	**38.7 (23.9 to 53.5)**	4.11 (–9.21 to 17.4)	18.4 (–8.50 to 45.3)
Difference in the step changes in monthly rates per 100,000 immediately following the policy changes (opioid v control group)	5.42 (–5.28 to 16.1)	16.9 (–3.71 to 37.4)	2.09 (–12.7 to 16.9)	11.4 (–30.4 to 53.2)
Trends change (slope) (control group)	0.22 (–0.16 to 0.60)	–1.03 (–2.06 to 0.00)	–0.60 (–1.22 to 0.01)	**–2.69 (–4.72 to –0.66)**
Trends change (slope) (opioid group)	0.06 (–0.64 to 0.77)	–0.53 (–1.78 to 0.72)	0.47 (–0.44 to 1.39)	–0.33 (–2.38 to 1.71)
Difference in changes in trends (slope) before and after the policy changes (opioid v control group)	–0.15 (–0.95 to 0.64)	0.50 (–1.13 to 2.13)	1.07 (–0.00 to 2.15)	2.36 (–0.51 to 5.22)
Level change at the end study period [a] (opioid group)	3.34 (–6.91 to 13.6)	–2.87 (–30.8 to 25.0)	–10.4 (–21.7 to 0.92)	**–51.1 (–90.1 to –12.0)**
Level change at the end study period (control group)	6.14 (–16.1 to 28.4)	24.3 (–5.32 to 53.9)	14.9 (–5.05 to 34.8)	9.70 (–43.1 to 62.5)
Difference in difference overall effect [b] (opioid v control group)	2.81 (–9.16 to 14.8)	27.2 (–29.6 to 28.9)	**25.3 (16.7 to 33.9)**	**60.8 (47.0 to 74.6)**
Post-intervention linear trend (control group)	–0.44 (–0.88 to 0.00)	**–1.81 (–2.84 to –0.77)**	–0.13 (–0.83 to 0.57)	–1.59 (–3.38 to 0.21)
Post-intervention linear trend (opioid group)	0.00 (–0.26 to 0.26)	**–1.05 (–1.80 to –0.30)**	**–0.57 (–1.00 to –0.13)**	**–2.03 (–3.60 to –0.45)**

[a] Level change at end of study period and predicted level from control period trends.

[b.] Difference between level at end of study period and predicted level from control period trends.

Blue: results included in Box 3 of main article.

Table 6. Interrupted time-series analysis (ITSA) results in ED presentation and hospital admissions before and after implementation of opioid policies between the opioid group and the control group (sensitivity analysis)

	**Coefficient (β) (95% confidence interval)**			
**Characteristic**	**Opioid-related**	**Non-opioid substance-related**	**Self-harm-related**	**Mental health-related**
**Emergency department presentations**				
Pre-intervention				
Difference in the initial step of monthly rates per 100,000 Opioid v control group	4.62 (–0.02 to 9.27)	10.6 (–3.78 to 24.89)	6.17 (–2.59 to 14.9)	16.0 (–0.22 to 32.2)
Difference in the trends (slope) Opioid v control group	0.36 (–0.02 to 0.74)	–0.59 (–1.60 to 0.43)	0.02 (–0.67 to 0.71)	–1.15 (–2.56 to 0.27)
Post-intervention				
Difference in the step changes in monthly rates per 100,000 immediately following the policy changes Opioid group v control group	–3.54 (12.1 to 5.02)	15.0 (–2.16 to 32.2)	0.66 (–15.1 to 16.4)	13.5 (–14.5 to 41.5)
Difference in changes in trends (slope) before and after the policy changes Opioid group v control group	–0.48 (–1.05 to 0.09)	–0.11 (–1.48 to1.26)	–0.33 (–1.45 to 0.79)	1.50 (–0.41 to 3.40)
**Hospital admissions**				
Pre-intervention	–0.31 (–0.67 to 0.05)	–0.020 (–0.79 to 0.75)	–0.16 (–0.51 to 0.19)	0.62 (–1.50 to 2.74)
Difference in the initial step of monthly rates per 100,000 Opioid v control group	**26.2* (14.6 to 37.9)**	**60.5* (46.2 to 74.8)**	**24.3* (17.7 to 31.0)**	**104* (62.6 to 145)**
Difference in the trends (slope) Opioid v control group	–0.25 (–1.20 to 0.70)	**–2.06* (–3.08 to –1.04)**	**–0.73* (–1.22 to –0.24)**	–1.70 (–4.53 to1.14)
Post-intervention				
Difference in the step changes in monthly rates per 100,000 immediately following the policy changes Opioid v control group	4.90 (–11.0 to 21.0)	19.2 (–6.19 to 44.7)	4.59 (–9.99 to 19.2)	–2.10 (–62.7 to 58.5)
Difference in changes in trends (slope) before and after the policy changes Opioid v control group	–0.12 (–1.24 to 1.00)	1.81 (–0.17 to 3.78)	0.91 (–0.24 to 2.06)	1.92 (–2.38 to 6.23)

References

1. Tsang RS, Joy M, Whitaker H, et al. Development of a modified Cambridge Multimorbidity Score for use with SNOMED CT: an observational English primary care sentinel network study. *Br J Gen Pract* 2023; 73: e435-e442.
2. Linden A. Conducting interrupted time-series analysis for single-and multiple-group comparisons. Stata J 2015; 15: 480-500.
3. Jebb AT, Tay L, Wang W, et al. Time series analysis for psychological research: examining and forecasting change. *Front Psychol* 2015; 6: 727.
4. Collyer TA, Athanasopoulos G, Srikanth V, et al. Impact of COVID-19 lockdowns on hospital presentations and admissions in the context of low community transmission: evidence from time series analysis in Melbourne, Australia. J Epidemiol Community Health 2022; 76: 341-349.

The RECORD statement. checklist of items, extended from the STROBE statement, that should be reported in observational studies using routinely collected health data

Note: The page and section numbers refer to the submitted manuscript, not the published article or its supporting information file.

	**Item No.**	**STROBE items**	**Location in manuscript where items are reported**	**RECORD items**	**Location in manuscript where items are reported**
**Title and abstract**					
	1	(a) Indicate the study's design with a commonly used term in the title or the abstract (b) Provide in the abstract an informative and balanced summary of what was done and what was found		RECORD 1.1: The type of data used should be specified in the title or abstract. When possible, the name of the databases used should be included.<br><br>RECORD 1.2: If applicable, the geographic region and timeframe within which the study took place should be reported in the title or abstract.<br><br>RECORD 1.3: If Page between databases was conducted for the study, this should be clearly stated in the title or abstract.	Title and abstract<br><br>Abstract<br><br>Title and abstract
**Introduction**					
Background rationale	2	Explain the scientific background and rationale for the investigation being reported			Introduction paragraphs 1-3
Objectives	3	State specific objectives, including any prespecified hypotheses			Introduction paragraphs 4-5
**Methods**					
Study Design	4	Present key elements of study design early in the paper			Method Design and setting
Setting	5	Describe the setting, locations, and relevant dates, including periods of recruitment, exposure, follow-up, and data collection			Method Design and setting
Participants	6	*(a) Cohort study* - Give the eligibility criteria, and the sources and methods of selection of participants. Describe methods of follow-up *Case-control study* - Give the eligibility criteria, and the sources and methods of case ascertainment and control selection. Give the rationale for the choice of cases and controls *Cross-sectional study* - Give the eligibility criteria, and the sources and methods of selection of participants<br><br>*(b) Cohort study* - For matched studies, give		RECORD 6.1: The methods of study population selection (such as codes or algorithms used to identify subjects) should be listed in detail. If this is not possible, an explanation should be provided.<br><br>RECORD 6.2: Any validation studies of the codes or algorithms used to select the population should be referenced. If validation was conducted for this study and not published elsewhere, detailed methods and results should be provided.<br><br>RECORD 6.3: If the study involved linkage of databases, consider use of a flow diagram	Method Population<br><br>Supplementary TableS1

		matching criteria and number of exposed and unexposed<br>*Case-control study* - For matched studies, give matching criteria and the number of controls per case		or other graphical display to demonstrate the data linkage process, including the number of individuals with linked data at each stage.	Method<br>Data source
Variables	7	Clearly define all outcomes, exposures, predictors, potential confounders, and effect modifiers. Give diagnostic criteria, if applicable.		RECORD 7.1: A complete list of codes and algorithms used to classify exposures, outcomes, confounders, and effect modifiers should be provided. If these cannot be reported, an explanation should be provided.	Method<br>Population<br><br>-opioid group<br>- propensity score matched controls
Data sources/ measurem ent	8	For each variable of interest, give sources of data and details of methods of assessment (measurement).<br>Describe comparability of assessment methods if there is more than one group			Method<br>Population<br><br>-opioid group<br>- propensity score matched controls<br>Supplementar y TableS3
Bias	9	Describe any efforts to address potential sources of bias			Method<br>Population<br><br>propensity score matched controls.
Study size	10	Explain how the study size was arrived at			NA
Quantitati ve variables	11	Explain how quantitative variables were handled in the analyses. If applicable, describe which groupings were chosen, and why			NA
Statistical methods	12	(a) Describe all statistical methods, including those used to control for confounding<br>(b) Describe any methods used to examine subgroups and interactions<br>(c) Explain how missing data were addressed<br>(d) *Cohort study* - If applicable, explain how loss to follow-up was addressed<br>*Case-control study* - If applicable, explain how matching of cases and controls was addressed<br>*Cross-sectional study* - If applicable, describe analytical methods taking account of sampling strategy<br>(e) Describe any sensitivity analyses			Method<br>Statistical analysis

Data access and cleaning methods		..		RECORD 12.1: Authors should describe the extent to which the investigators had access to the database population used to create the study population.<br><br>RECORD 12.2: Authors should provide information on the data cleaning methods used in the study.	
Linkage		..		RECORD 12.3: State whether the study included person-level, institutional-level, or other data linkage across two or more databases. The methods of linkage and methods of linkage quality evaluation should be provided.	Method<br>Data source
**Results**					
Participants	13	(a) Report the numbers of individuals at each stage of the study (*e.g.*, numbers potentially eligible, examined for eligibility, confirmed eligible, included in the study, completing follow-up, and analysed)<br>(b) Give reasons for non-participation at each stage.<br>(c) Consider use of a flow diagram		RECORD 13.1: Describe in detail the selection of the persons included in the study (*i.e.,* study population selection) including filtering based on data quality, data availability and linkage. The selection of included persons can be described in the text and/or by means of the study flow diagram.	Results<br>Paragraph 1
Descriptive data	14	(a) Give characteristics of study participants (*e.g.*, demographic, clinical, social) and information on exposures and potential confounders<br>(b) Indicate the number of participants with missing data for each variable of interest<br>(c) *Cohort study* - summarise follow-up time (*e.g.*, average and total amount)			Supplementary TableS4
Outcome data	15	*Cohort study* - Report numbers of outcome events or summary measures over time<br>*Case-control study* - Report numbers in each exposure category, or summary measures of exposure<br>*Cross-sectional study* - Report numbers of outcome events or summary measures			Results<br>Figure 1&2<br>Table 1
Main results	16	(a) Give unadjusted estimates and, if applicable, confounder-adjusted estimates and their precision (e.g., 95% confidence interval). Make clear which confounders were			Results<br>Figure 1&2<br>Table 1

		adjusted for and why they were included<br>(b) Report category boundaries when continuous variables were categorized<br>(c) If relevant, consider translating estimates of relative risk into absolute risk for a meaningful time period			
Other analyses	17	Report other analyses done—e.g., analyses of subgroups and interactions, and sensitivity analyses			Supplementary TableS5
**Discussion**					
Key results	18	Summarise key results with reference to study objectives			Discussion Paragraph 1
Limitations	19	Discuss limitations of the study, taking into account sources of potential bias or imprecision. Discuss both direction and magnitude of any potential bias		RECORD 19.1: Discuss the implications of using data that were not created or collected to answer the specific research question(s). Include discussion of misclassification bias, unmeasured confounding, missing data, and changing eligibility over time, as they pertain to the study being reported.	Discussion Strengths and limitations
Interpretation	20	Give a cautious overall interpretation of results considering objectives, limitations, multiplicity of analyses, results from similar studies, and other relevant evidence			Discussion Paragraph 2-3
Generalisability	21	Discuss the generalisability (external validity) of the study results			Discussion Strengths and limitations
**Other Information**					
Funding	22	Give the source of funding and the role of the funders for the present study and, if applicable, for the original study on which the present article is based			Discussion Strengths and limitations
Accessibility of protocol, raw data, and programming code		..		RECORD 22.1: Authors should provide information on how to access any supplemental information such as the study protocol, raw data, or programming code.	**Data statement**

*Reference: Benchimol EI, Smeeth L, Guttmann A, Harron K, Moher D, Petersen I, Sørensen HT, von Elm E, Langan SM, the RECORD Working Committee. The REporting of studies Conducted using Observational Routinely-collected health Data (RECORD) Statement. *PLoS Medicine* 2015; 015 Oct 6;12(10):e1001885.

*Checklist is protected under Creative Commons Attribution (CC BY) license.
